# YOLO with Kolmogorov-Arnold networks and vision-language foundation models for interpretable object detection with trustworthy multimodal AI in computer vision perception

**DOI:** 10.1038/s41598-026-57596-x

**Published:** 2026-06-16

**Authors:** Marios Impraimakis, Daniel Vazquez, Feiyu Zhou

**Affiliations:** 1 Department of Mechanical Engineering, University of Bath, Bath, BA2 7AY UK; 2 Department of Civil Engineering, Zhejiang University, Hangzhou, 310027 China

**Keywords:** Trustworthy object detection, Kolmogorov-Arnold Network (KAN), You Only Look Once (YOLOv10), Interpretable Explainable AI (XAI), Engineering, Mathematics and computing

## Abstract

The trustworthy object detection capabilities of a novel Kolmogorov-Arnold network framework are examined here. The approach addresses a key limitation in computer vision for vehicle detection perception, and beyond. These systems offer limited transparency regarding the reliability of their confidence scores in visually degraded or ambiguous scenes. To this end, a Kolmogorov-Arnold network is employed as an interpretable post-hoc surrogate to model the trustworthiness of the You Only Look Once (Yolov10) detections using seven geometric and semantic features. The additive spline-based structure of the Kolmogorov-Arnold network enables direct visualisation of each feature’s influence. This produces smooth and transparent functional mappings that reveal when the model’s confidence is well supported and when it is unreliable. Furthermore, a bootstrapped language-image (BLIP) foundation model generates descriptive captions of each scene. This tool enables a lightweight multimodal interface without affecting the interpretability layer. Experiments on both Common Objects in Context (COCO), and images from the University of Bath campus demonstrate that the framework accurately identifies low-trust predictions under blur, occlusion, or low texture. This provides actionable insights for acceptance, review, or downstream risk mitigation. The resulting system delivers interpretable object detection with trustworthy confidence estimates. It offers a powerful tool for transparent and practical perception component for autonomous and multimodal artificial intelligence applications.

Object detection systems have attracted interest through the development of the one-stage You Only Look Once models. These detectors achieve real-time performance, making them attractive for deployment in visual perception tasks in autonomous vehicles^1–4^, traffic^5^, pedestrian detection^6–8^, and object tracking^9–13^. Other areas include material images^14–16^, or medical applications^17,18^.

To this end, Wang et al.^19^ proposed to train the You Only Look Once models^20–25^ without a filtering step for faster and more accurate behavior. Dong et al.^26^ developed a method that uses wavelet-based processing, improved feature pooling, and a combined loss design. Later, Luz et al.^27^ showed that combining Internet of Things devices, edge computing, and deep learning enables vehicle detection across hardware with widely varying processing speeds. Furthermore, Wang et al.^28^ improved the detection of tiny objects by mixing information from different scales and surrounding context^29^. For materials, Haoyan et al.^30^ proposed a lightweight model for steel-surface defect detection by replacing standard layers with an adaptive downsampling structure^31,32^. Chhimpa et al.^33^, later, demonstrated that fine-tuning an object detection model can effectively identify brain tumors in medical images. Furthermore, An et al.^34^ introduced a small-target model which improves crack detection using a new head structure, a detail-enhancing pyramid, and a loss function. Zhang et al.^35^ also proposed an improved network specifically for aerial vehicle detection, Ding et al.^36^ developed a model that uses attention and multi-scale processing to detect small objects from drone imagery, while Chen et al.^37^ added an edge-focused feature module, improved multi-scale processing, and pruning and distillation steps. For complex applications, Sun et al.^38^ also combined a transformer-based backbone with an attention-driven feature-fusion method to capture tiny details. Srinivasu et al.^39^ studied how adjusting model settings and expanding the training data affects the detection performance. Mahapadi et al.^40^ later optimized two nature-inspired search algorithms. Along these lines, Qin et al.^41^ proposed a classroom-behavior detection system which improves recognition under crowded and multi-scale conditions. Furthermore, Xie et al.^42^ described a model for autonomous-driving detection^43–49^ that uses a new gating module, an improved feature pyramid, a redesigned multi-scale network, and a double-distillation training strategy. In mechanical engineering, Liu et al.^50^ introduced an enhanced model designed to locate defects in aircraft-engine components. Finally, Meng et al.^51^ combine ideas from pose-estimation models for agricultural automation.

Regarding Kolmogorov-Arnold networks proposed by Liu et al.^52^, Shuai et al.^53^ introduced later a physics-informed framework that embeds scientific laws into the learning process^54–58^. Wu et al.^59^ presented Graph Kolmogorov-Arnold networks^60^, while Howard et al.^61^ developed multifidelity Kolmogorov-Arnold networks that use inexpensive low-quality data. Hasan et al.^62^ combined sequence-learning networks to analyze brain-signal time series, while Baravsin et al.^63^ conducted a study across over one hundred time-series datasets for real-world signals. Furthermore, Wang et al.^64^ proposed convolutional Kolmogorov-Arnold networks, which aim to create smaller and more interpretable intrusion-detection models. Saravani et al.^65^ compared Kolmogorov–Arnold networks with machine-learning approaches, Ansar et al.^66^ modified the loss functions of Kolmogorov-Arnold networks incorporating a correlation measure to improve performance, while Ta et al.^67^ introduced fully-composed Kolmogorov-Arnold networks that mix mathematical basis functions to learn complex relationships from low-dimensional inputs. Finally, Panahi et al.^68^ proposed a general discovery framework using Kolmogorov-Arnold networks that can uncover governing equations from data.

To the vision-language modeling end^69–73^, Xue et al.^74^ introduced a curated dataset, training procedures, and a family of large models that handle both images and text^75^. Yang et al.^76^ presented VisionZip, a method that keeps only the most useful visual tokens, Xu et al.^77^ proposed a model with chain-of-thought that performs its own multi-stage reasoning steps, while Vasu et al.^78^ introduced a new vision encoder that produces fewer visual tokens to greatly speed up processing. To this end, Deng et al.^79^ studied how vision-language models balance visual and textual information when they are given images and different kinds of text inputs, Zhou et al.^80^ introduced the first benchmark for both space and time in videos, while Shu et al.^81^ proposed video-extra-large which summarizes the content of each time segment. Deitke et al.^82^ presented newly created datasets, Zhan et al.^83^ also curated a large remote-sensing instruction dataset^84^, and Hu et al.^85^ created a high-quality remote-sensing image-captioning dataset for aerial images. Furthermore, Wu et al.^86^ explored segmenting farmland applications, Dai et al.^87^ investigated the interactions between people and their environments, and Xin et al.^88^ examined a decomposed three-dimensional encoder, an improved image-text alignment method, and a dual-stream projector for medical images with text descriptions.

Related to interpretable artificial intelligence in engineering^89–93^, Singh et al.^94^ introduced DiaXplain, a diabetes-diagnosis tool to deliver understandable results, and Roy et al.^95^ developed a system that emphasizes transparency for clinicians, while Yao et al.^96^ proposed a method that detects interpretable discharge states. Chudasama et al.^97^ introduced TrustKG, a knowledge-graph-based approach that improves the interpretability and reliability of hybrid medical systems. Along these lines, Gliner et al.^98^ demonstrated a tool for electrocardiogram images, and Song et al.^99^ analyzed climate and lightning data to understand which conditions increase the likelihood of forest fire-related lightning^100^. Rashid et al.^101^ presented an ensemble-based disease-detection method, while Salvi et al.^102^ combined uncertainty estimation with interpretability methods. Finally, Agrawal et al.^103^ explored explanation techniques to improve the clarity of model reasoning.

Related to trustworthy artificial intelligence, Chander et al.^104^ described a range of modern relevant techniques with Wirz et al.^105^ argued that trust in outputs depends both on how users perceive the system and on the specific decision situation. Cousineau et al.^106^ considered findings from interviews with developers showing that creating trustworthy artificial-intelligence systems is difficult due to immature standards, inconsistent regulations, unclear definitions, and limited practical tools. Stamboliev et al.^107^ analyzed European Union policy discourse and concluded that promoting trustworthy artificial intelligence serves as a shared idea that unites diverse political and industry stakeholders. Importantly, Reinhardt et al.^108^ examined the philosophical views of trust, Goisauf et al.^109^ provided an interdisciplinary analysis of trust, while Jannel et al.^110^ proposed the concept of trustability as a threshold that distinguishes appropriate trust in these systems. Finally, Parra et al.^111^ introduced the reason framework, which aims to integrate trustworthiness into the full life-cycle of future communication networks, and Moreno et al.^112^ proposed a design approach intended to help developers embed trustworthy principles into medical systems.

However, the internal behavior of such models remains difficult to interpret: the learned decision surfaces are highly nonlinear, their dependence on geometric and semantic features is opaque, and their confidence outputs offer limited insight into why detections are accepted, rejected, or assigned particular confidence levels. To this end, a framework that couples You Only Look Once with a Kolmogorov-Arnold networks is examined to model and interpret the internal confidence predictions of the detector. In this work, the Kolmogorov-Arnold network model is supervised using the original You Only Look Once confidence scores as the target signal, thereby learning a structured and interpretable mapping from detection features to predicted confidence. The focus is on the structured seven-dimensional set of interpretable features available at the output of the detection head: normalized bounding-box position, normalized size, predicted confidence, class index, and relative image scale. Additionally, natural language explanations offer complementary insight by aligning model outputs with human linguistic reasoning. The combination of these models results in a unified interpretability pipeline: You Only Look Once performs object detection, the Kolmogorov-Arnold network provides a transparent surrogate that exposes the structure of the model’s confidence function, and the visual-language model produces natural language descriptions for human review. This multimodal design^113,114^ brings together visual, numerical, and linguistic interpretability within a single coherent framework.

Specifically, You Only Look Once is a one-stage object detector designed to perform real-time bounding-box prediction that directly regresses object locations and class probabilities, seen in Fig. 1. The network receives an input image of size $640 \times 640 \times 3$ with approximately 8400 candidate detections. Features are used by the Kolmogorov-Arnold Network, seen as a framework in Fig. 2. The Kolmogorov-Arnold network seen in Fig. 3 is used as a surrogate confidence model with architecture width=[7,16,1], grid=5 and spline order=3 (cubic b-splines), meaning that the network receives seven interpretable numerical features derived from detections. This architecture directly enables the interpretability results. Large vision-language foundation models also aim to learn a joint representation between images and natural language, seen in Fig. 4.

**Fig. 1:**
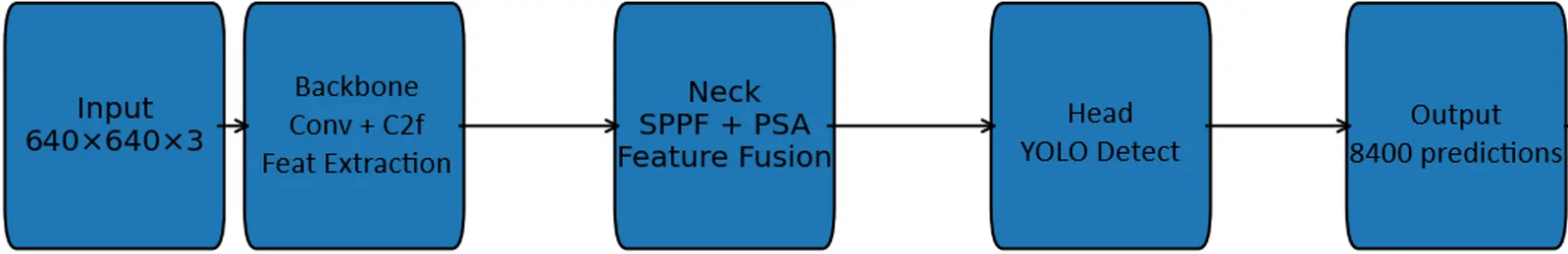
Architectural framework of You Only Look Once. C2f stands for coarse-to-fine, SPPF stands for spatial pyramid pooling fast, and PSA stands for partial self-attention.

**Fig. 2:**
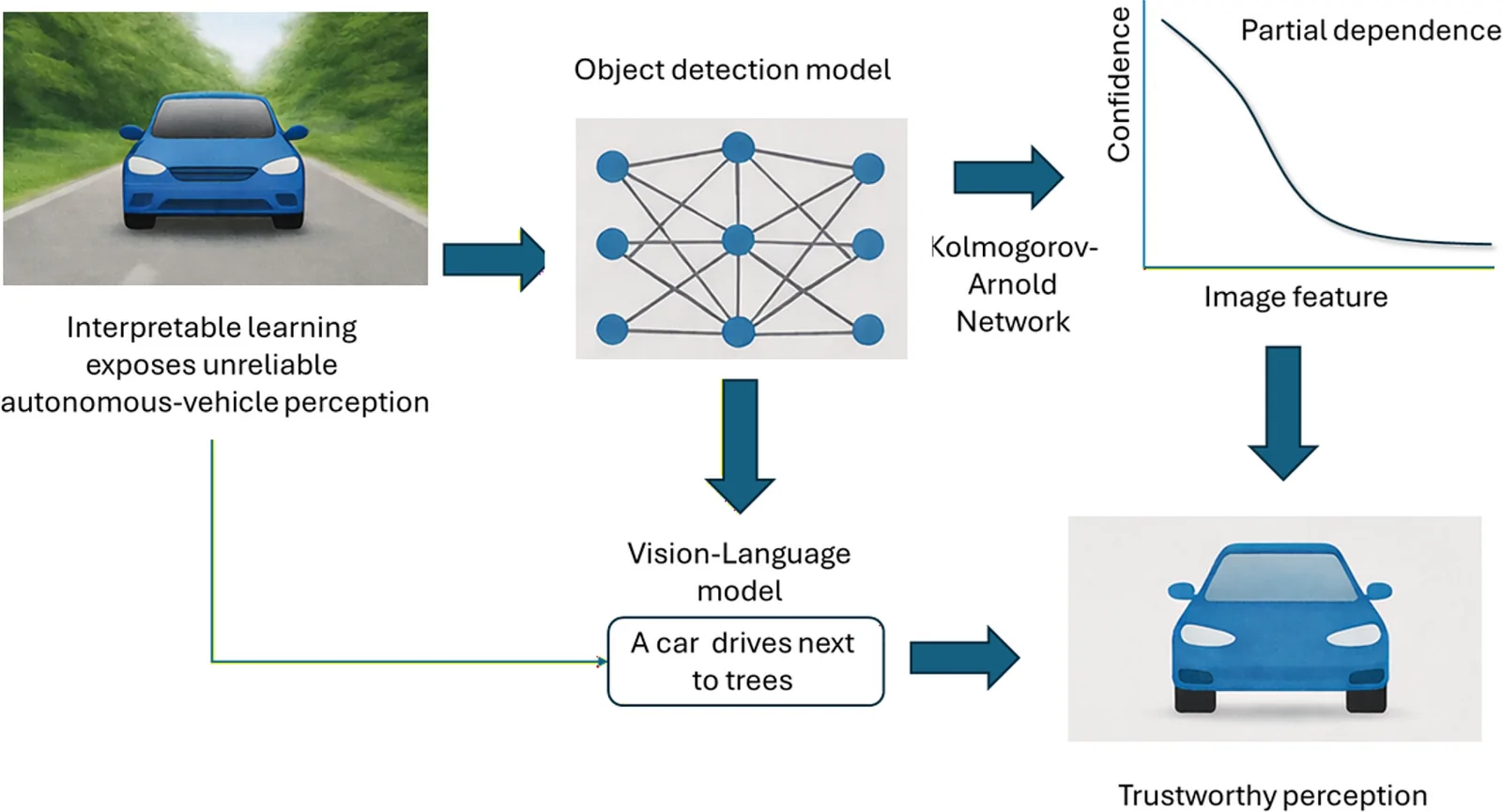
Proposed framework for the computer vision perception.

**Fig. 3:**
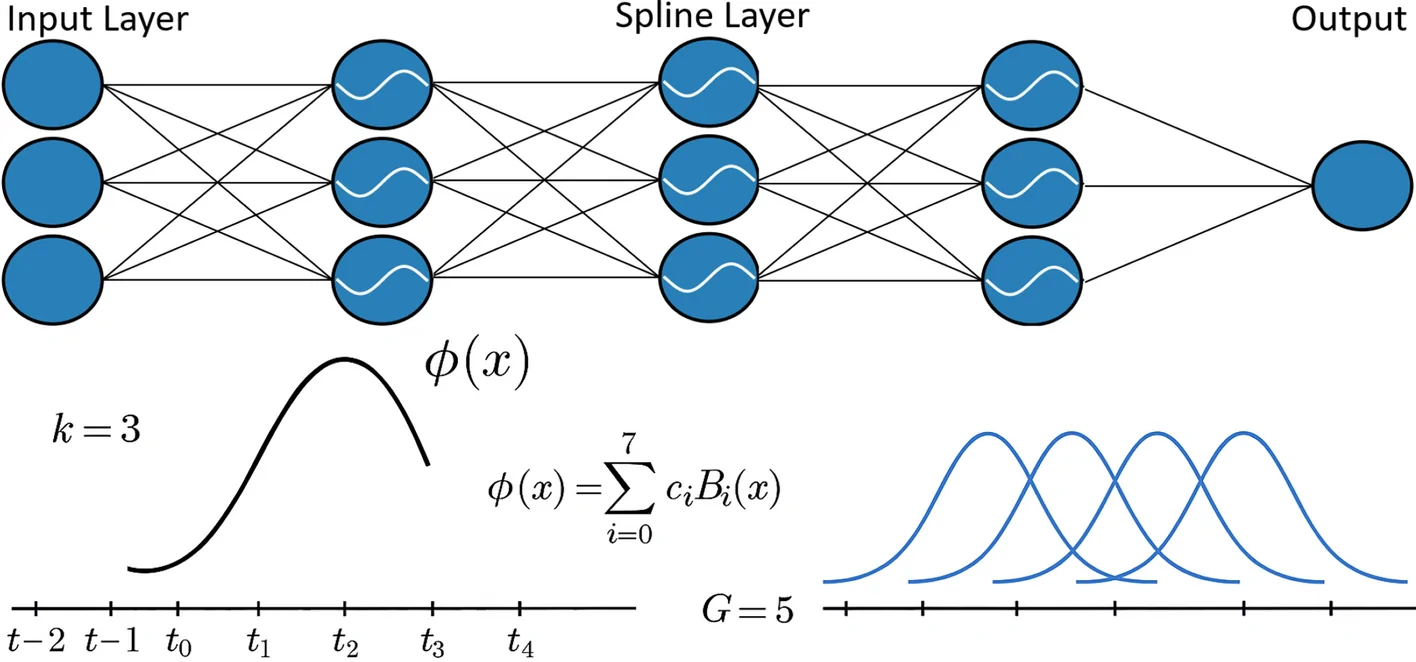
Architecture of a Kolmogorov-Arnold network.

**Fig. 4:**
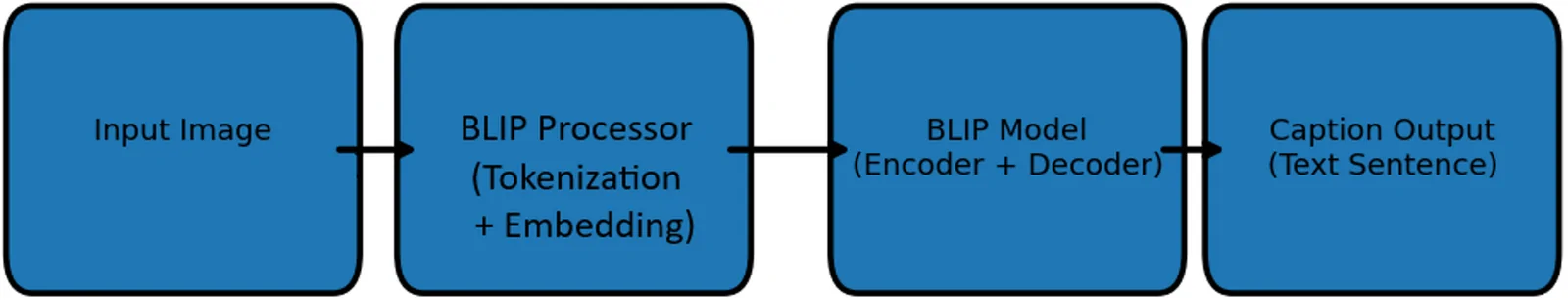
Architectural framework of visual-language models. BLIP stands for Bootstrapped Language-Image Pretraining.

The remainder of this paper is organized as follows. Section Results presents the main results including the feature-level analyses, partial-dependence behaviour, hidden-unit specialization, edge-importance structure, fidelity evaluation, and qualitative interpretability examples on both the COCO dataset and University of Bath images. Section Discussion provides a discussion of the interpretability findings, and concludes the work. Section Methods describes the methodological foundations, detailing the You Only Look Once confidence formulation, the Kolmogorov-Arnold network architecture and spline-based representation, and the vision-language model used to generate descriptive captions.

## Results

**Fig. 5:**
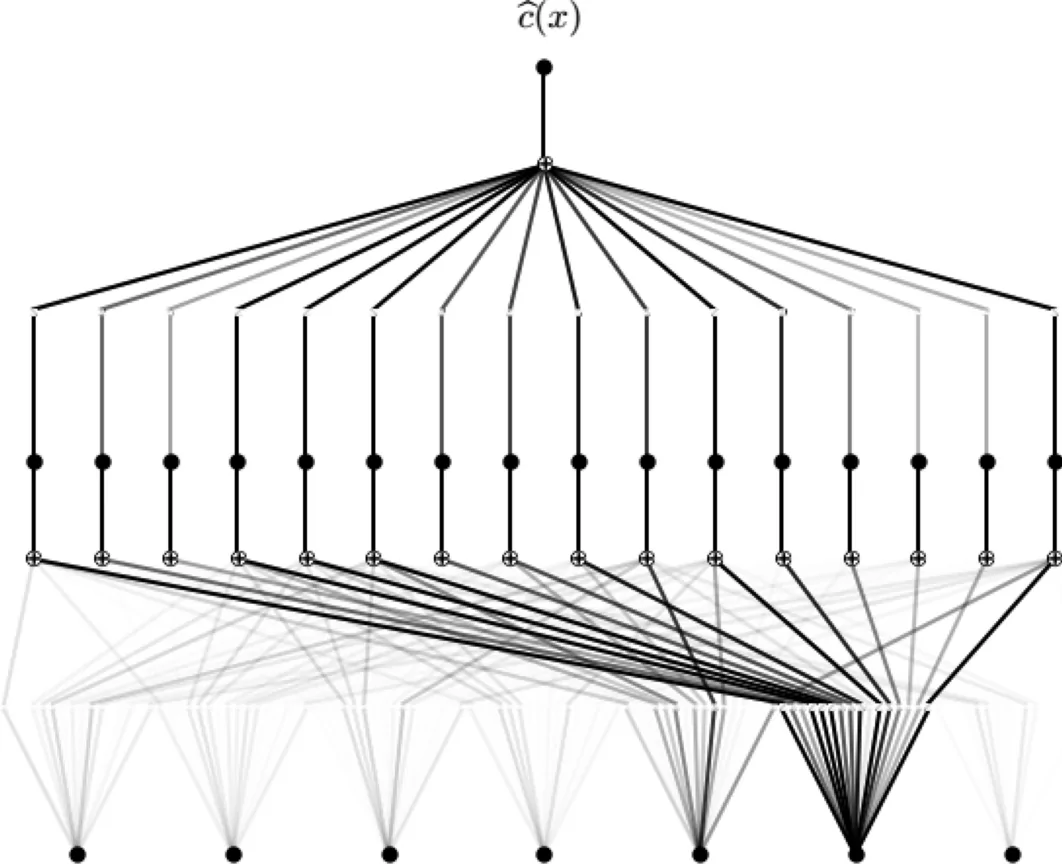
Kolmogorov-Arnold network model architecture with seven interpretable inputs and one output.

**Fig. 6:**
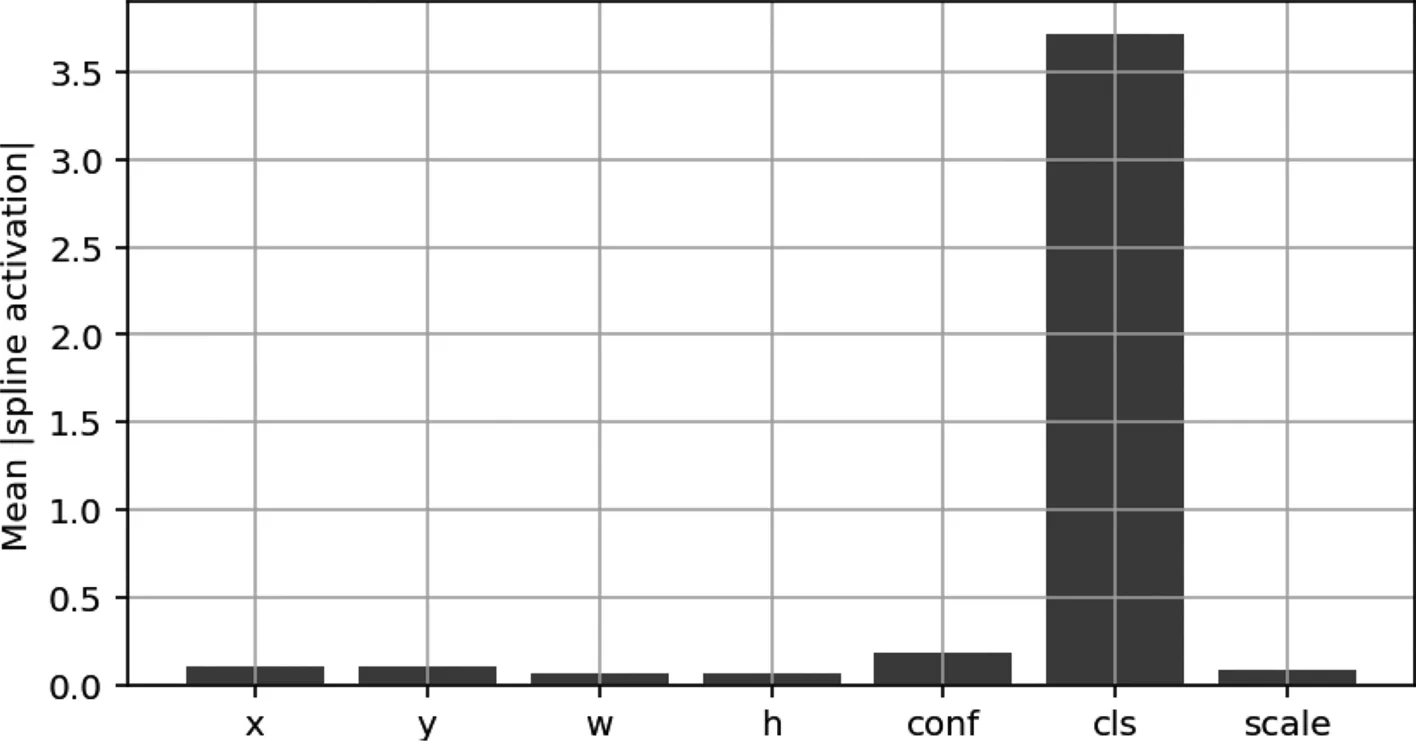
Mean absolute spline activation per input feature.

The modeling framework is examined on the Common Objects in COntext dataset focused on vehicle detection tasks. The goal is to characterize the model’s internal confidence behavior using the Kolmogorov-Arnold network, and to demonstrate how the spline-based structure provides transparent explanations of the detector’s geometric and semantic sensitivities. Here, You Only Look Once confidence scores are used as the target signal for Kolmogorov-Arnold network. The model, shown in Fig. 5, receives seven input features derived from You Only Look Once detections; bounding-box geometry, predicted confidence, class index, and image scale, and outputs a smooth approximation of the model’s confidence. The darker edges in the schematic indicate stronger learned importance. The mean absolute spline activation per input feature is shown in Fig. 6; here, the class index and confidence values dominate the activation magnitudes, while geometric terms (*x*, *y*, *w*, *h*) exhibit weaker but consistent influence.

**Fig. 7:**
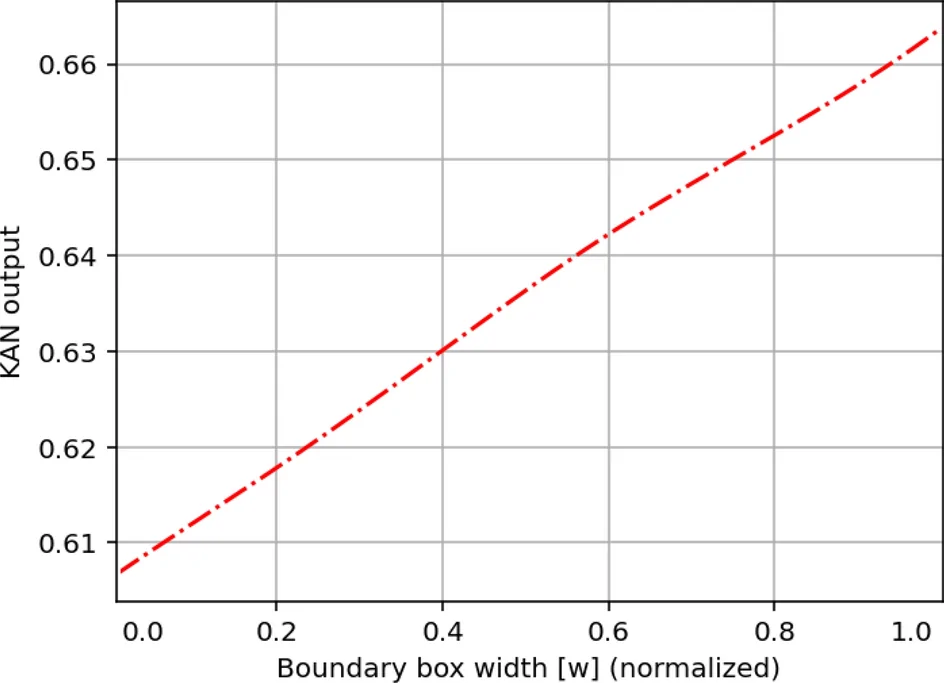
Partial dependence of the Kolmogorov-Arnold network on bounding-box width.

**Fig. 8:**
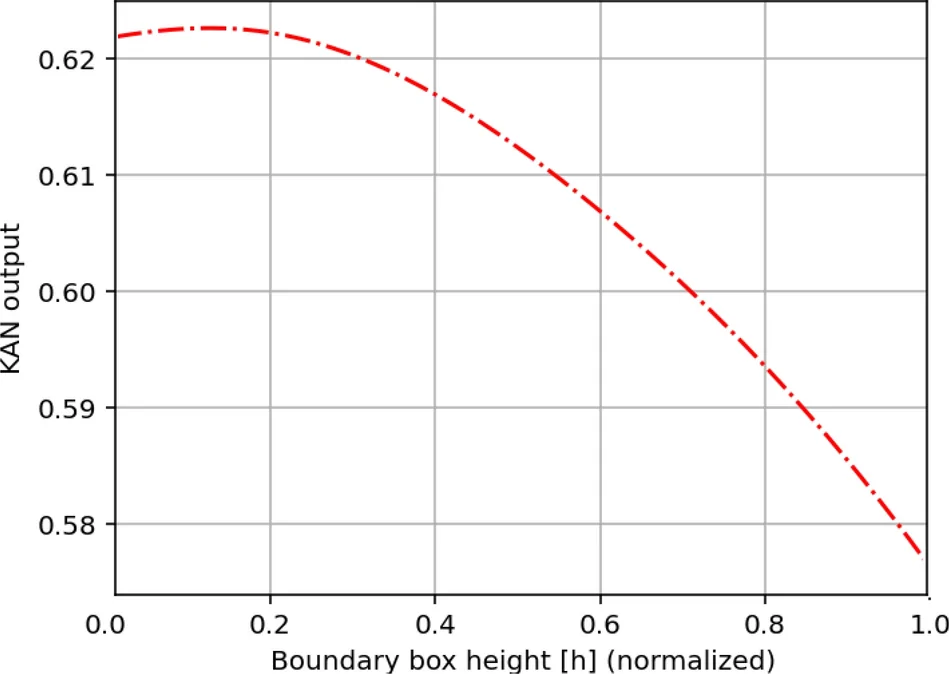
Partial dependence on bounding-box height.

**Fig. 9:**
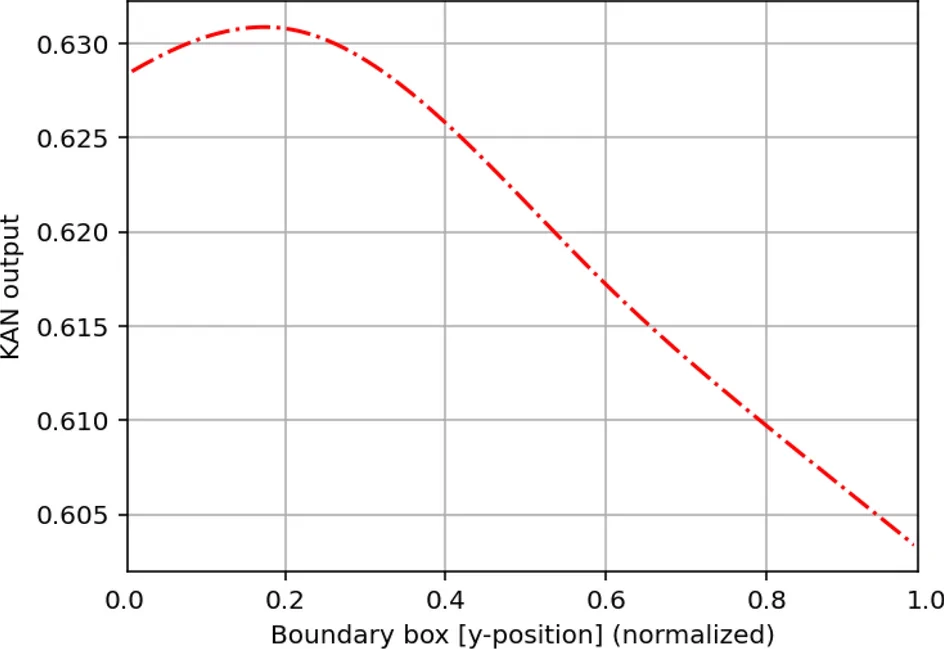
Partial dependence on vertical position *y*.

**Fig. 10:**
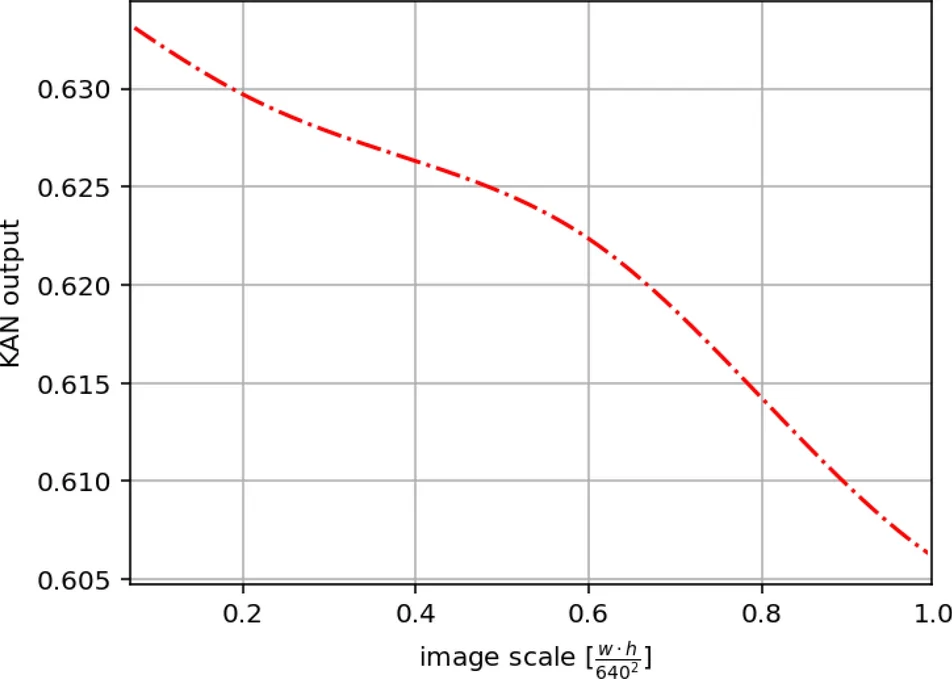
Partial dependence on image scale.

**Fig. 11:**
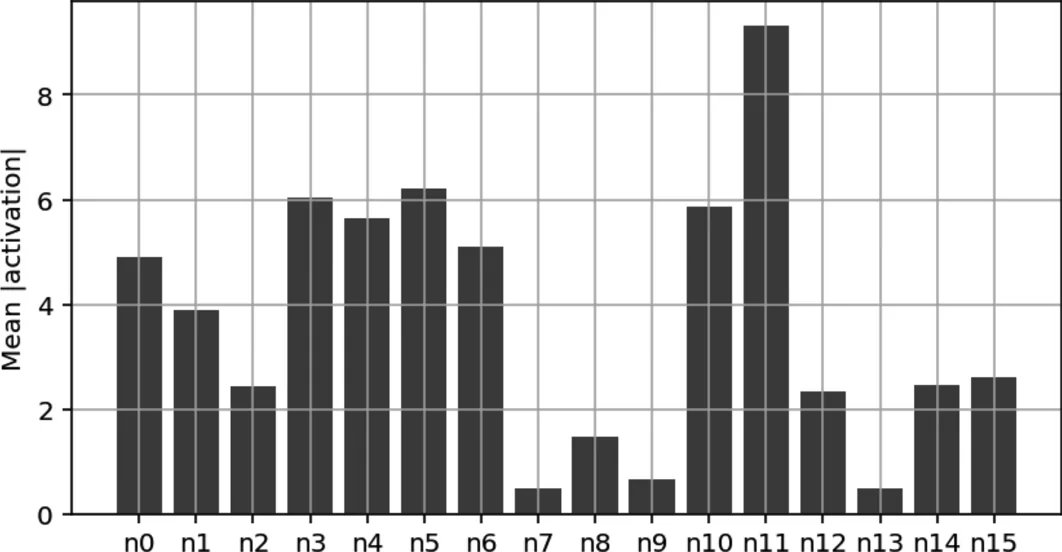
Feature influence on a representative hidden unit.

**Fig. 12:**
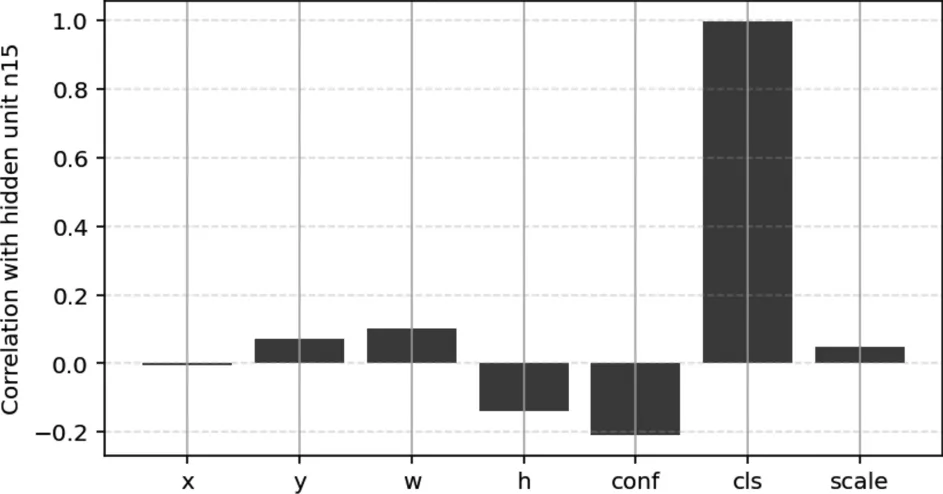
Feature influence on a different hidden unit.

**Fig. 13:**
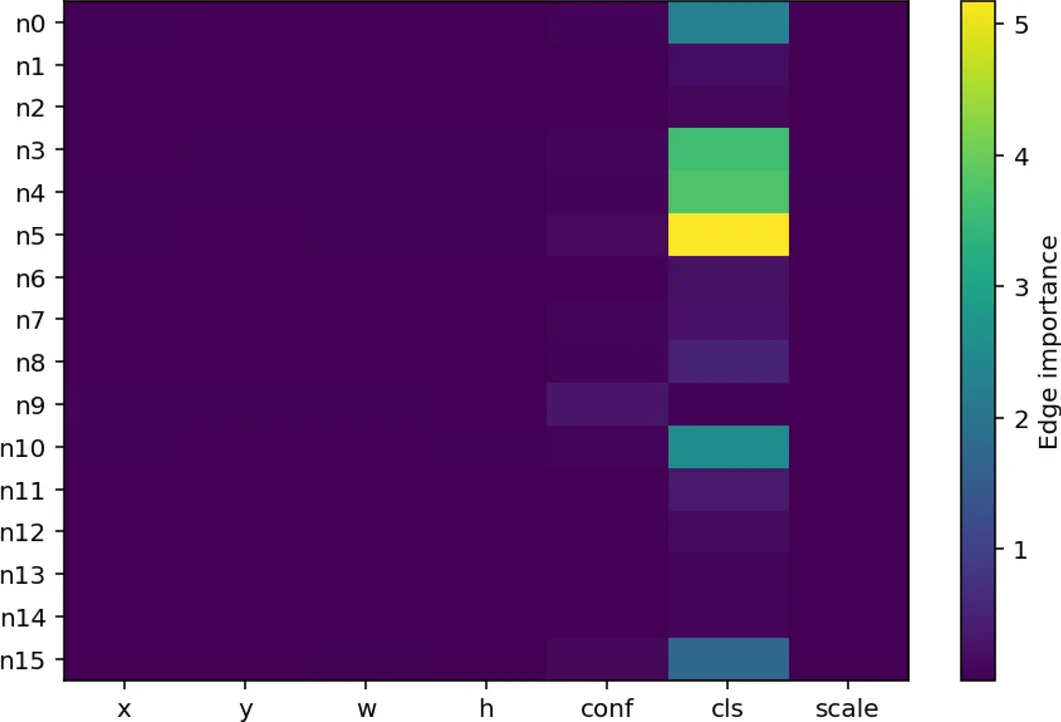
Input–hidden edge importance heatmap.

**Fig. 14:**
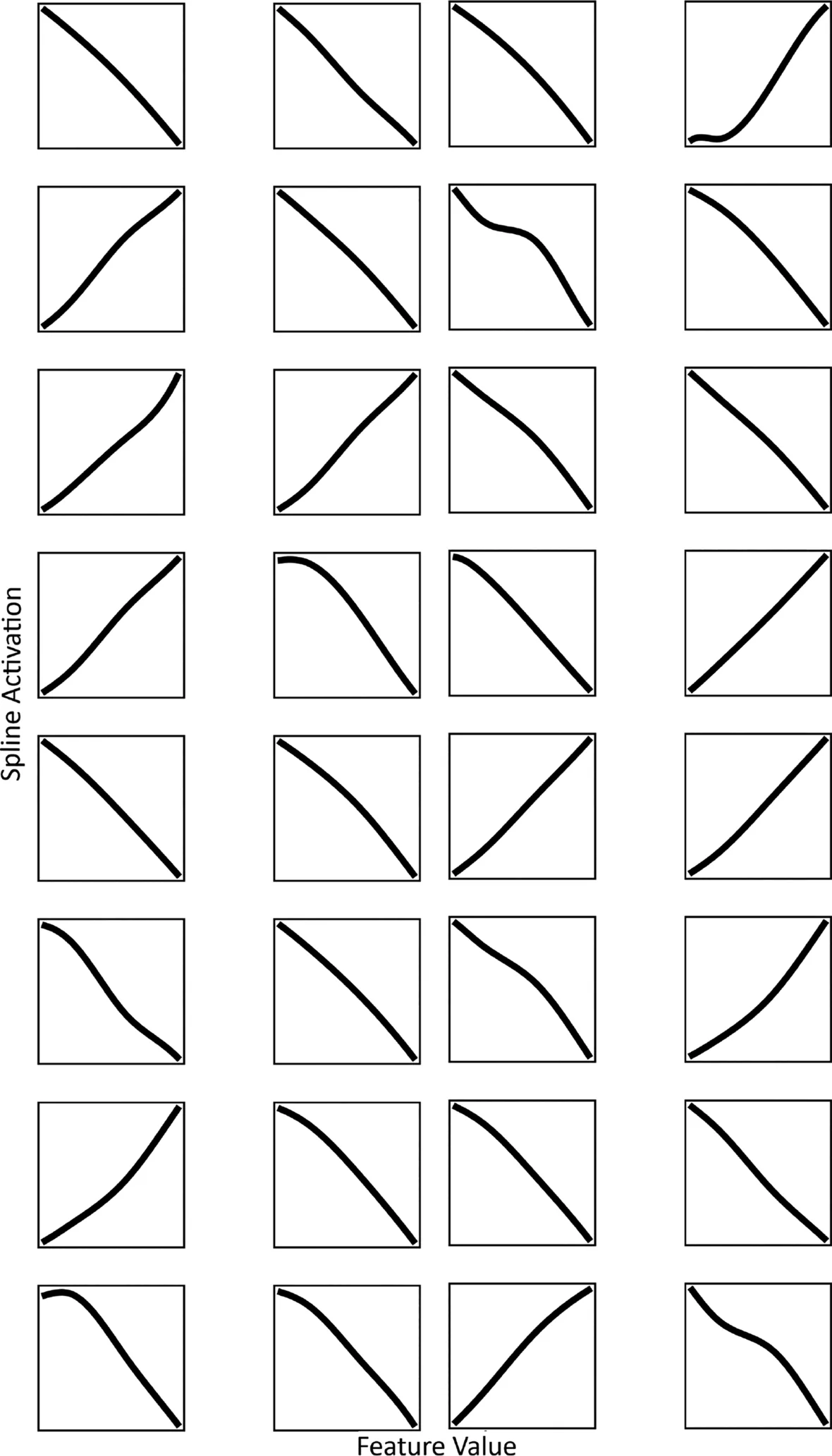
Spline functions for all feature - unit pairs (Group 1, horizontal-axis: feature value, vertical-axis: spline activation).

**Fig. 15:**
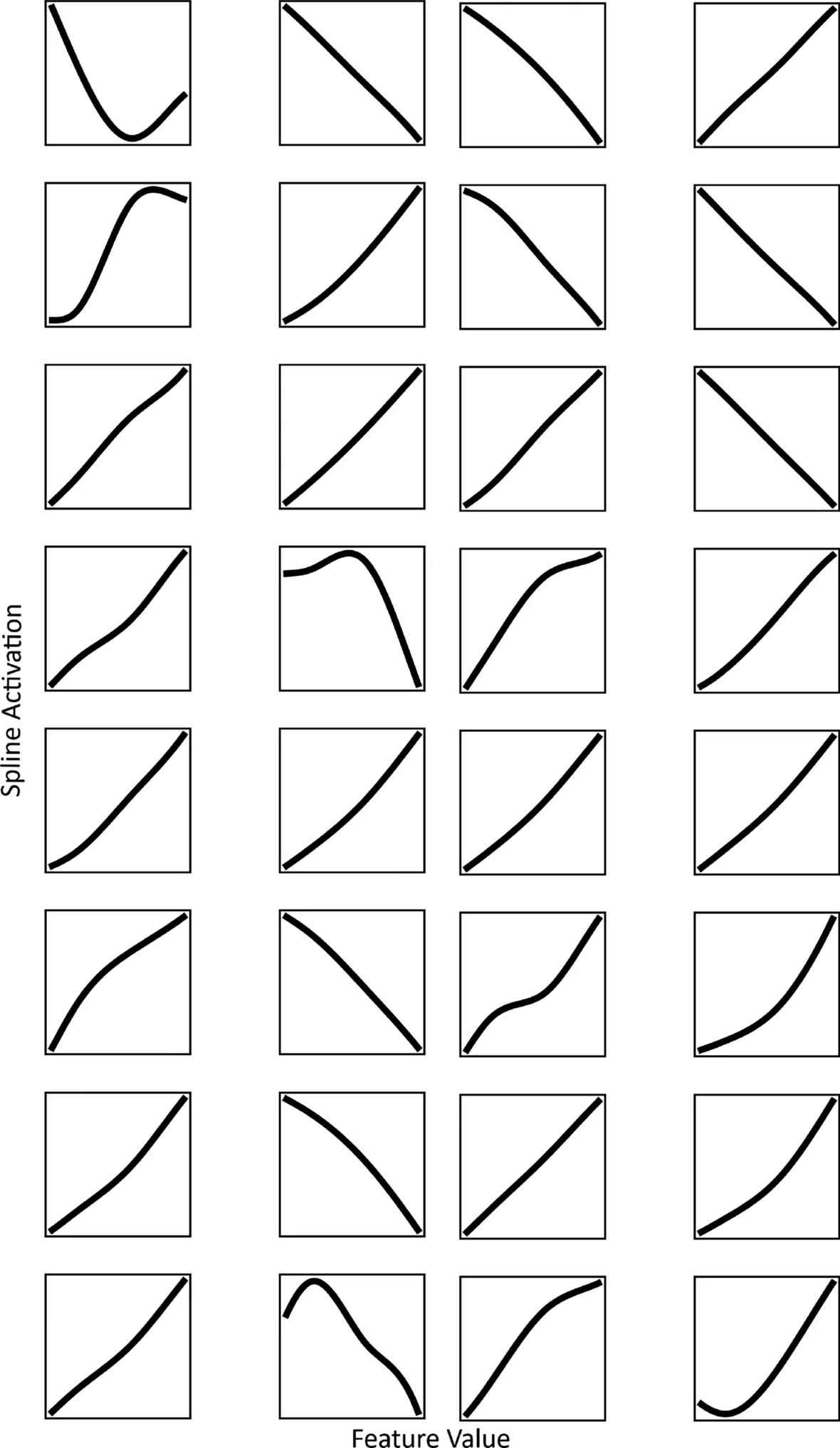
Spline functions for all feature - unit pairs (Group 2).

**Fig. 16:**
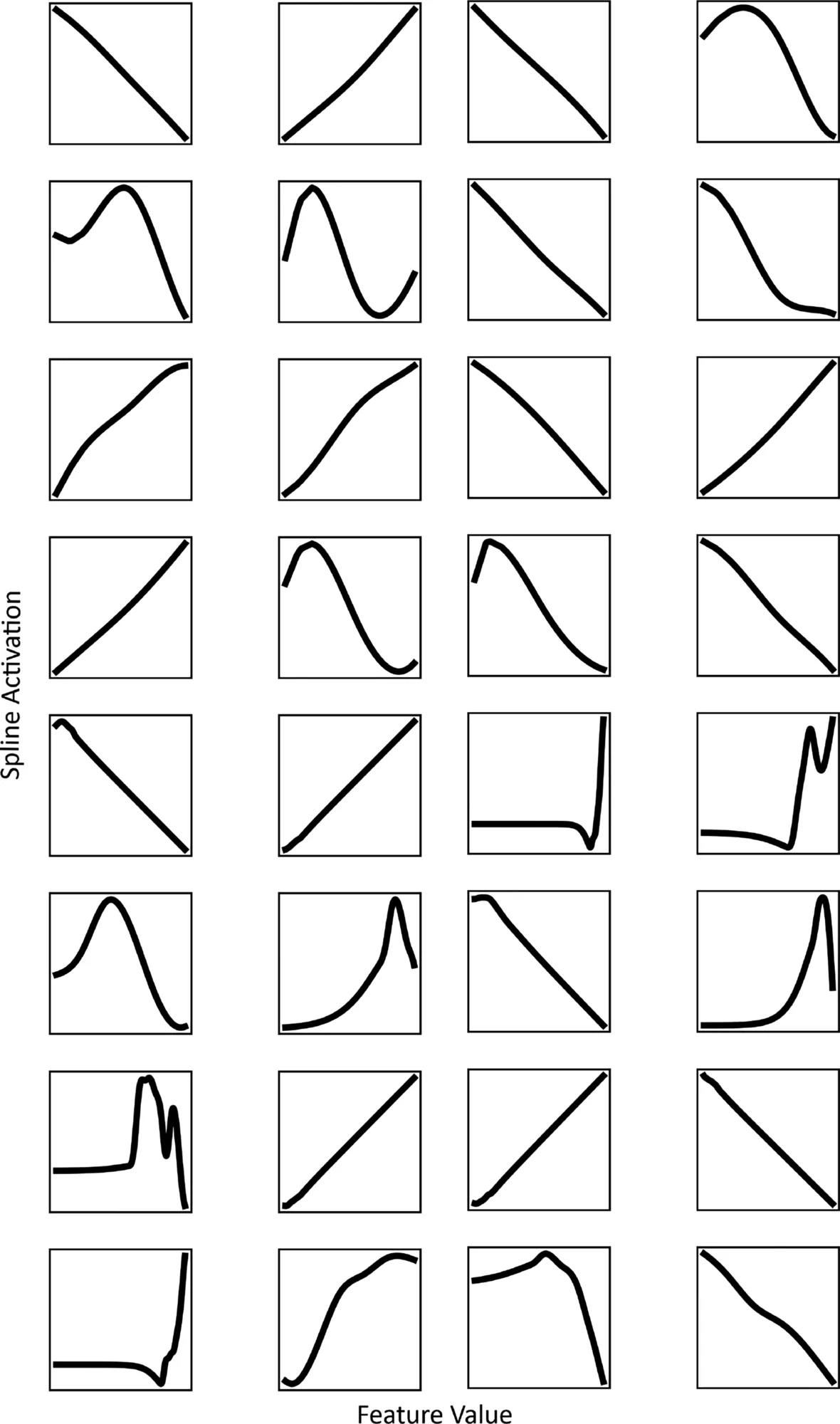
Spline functions for all feature - unit pairs (Group 3).

**Fig. 17:**
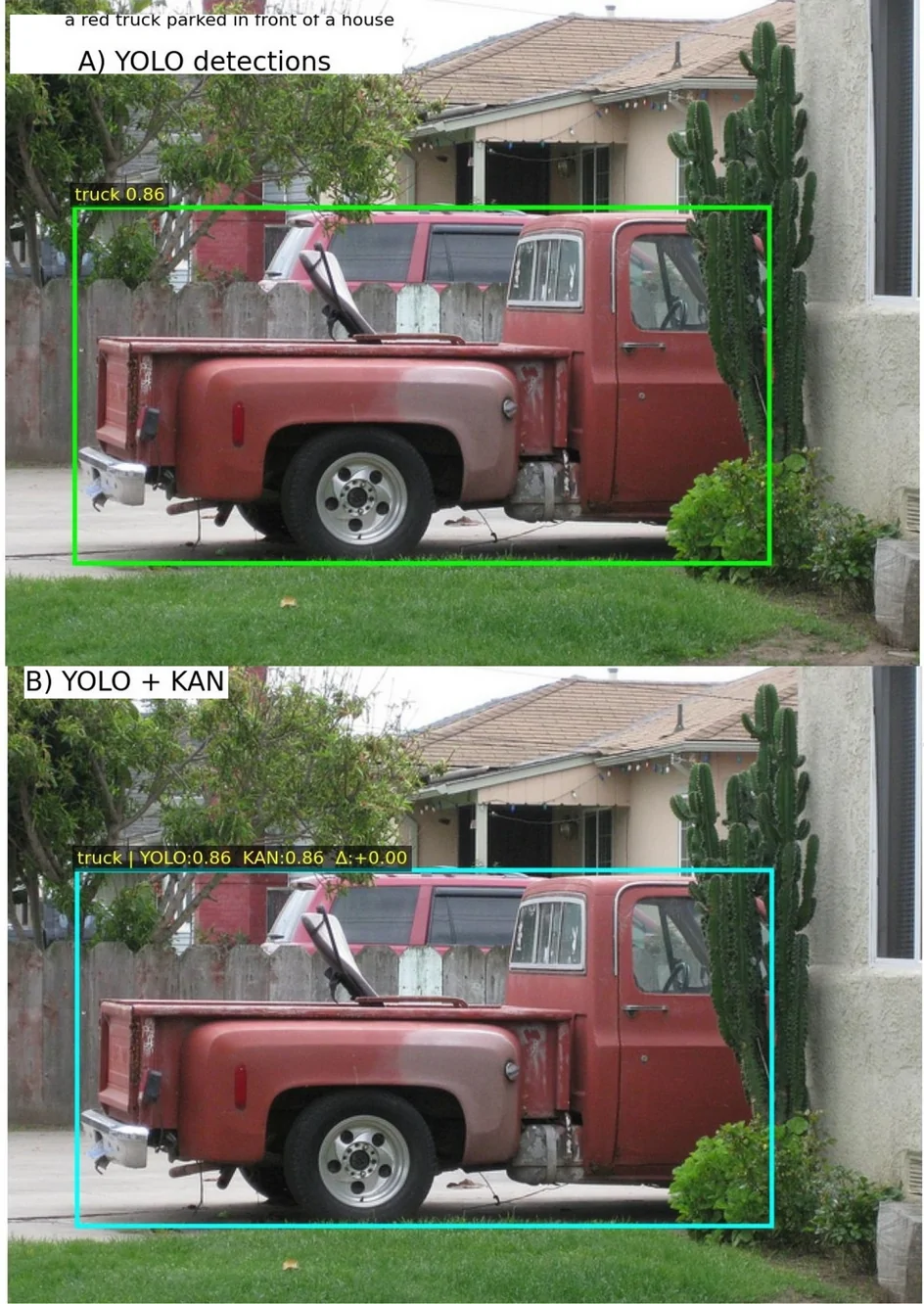
Application to a truck scene.

**Fig. 18:**
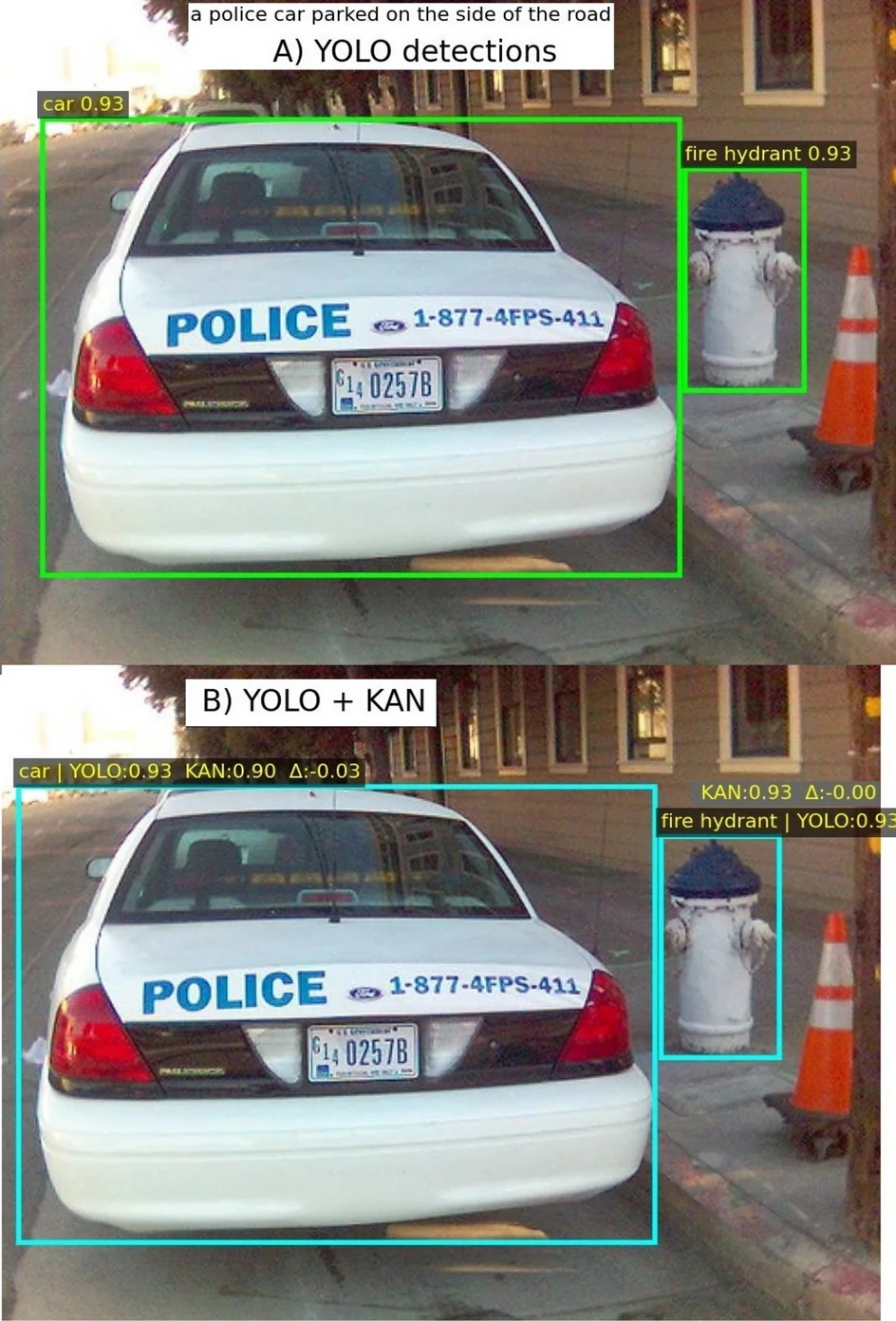
Application to a police car.

**Fig. 19:**
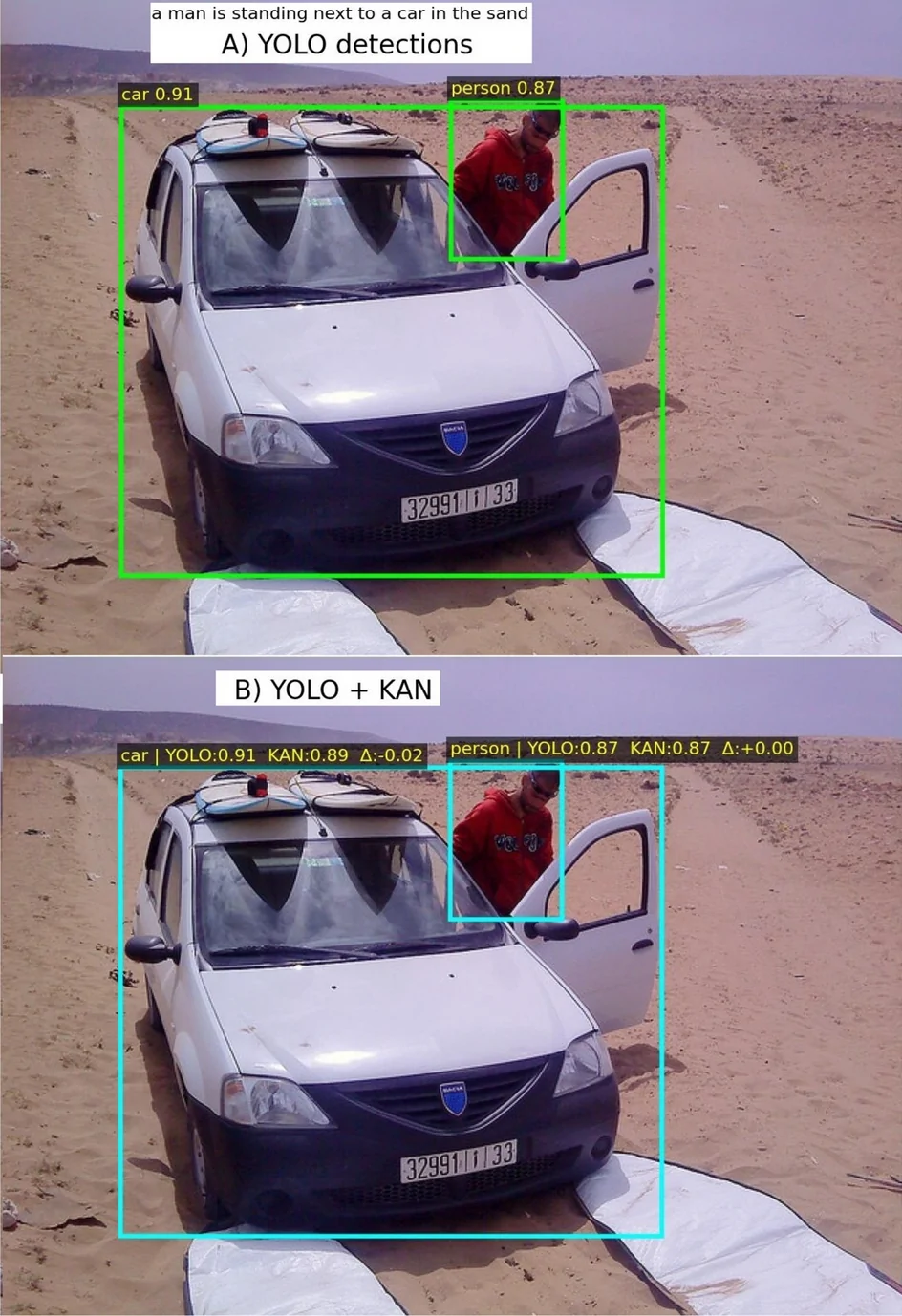
Application to a vehicle in sand. This image is from the Common Objects in Context dataset, available at https://cocodataset.org. All human images appearing in this dataset are provided under its licensing terms.

**Fig. 20:**
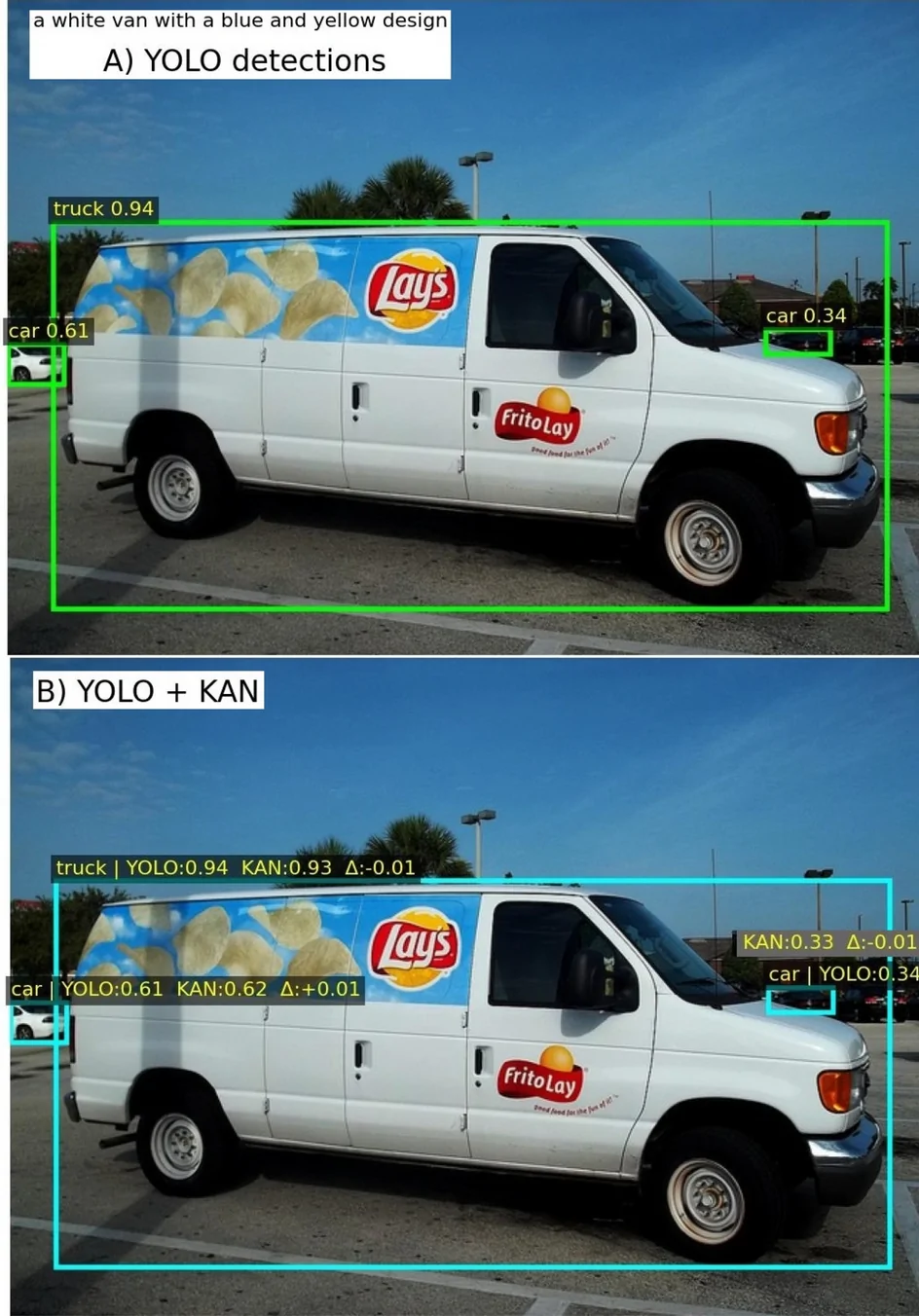
Application to a van example.

**Fig. 21:**
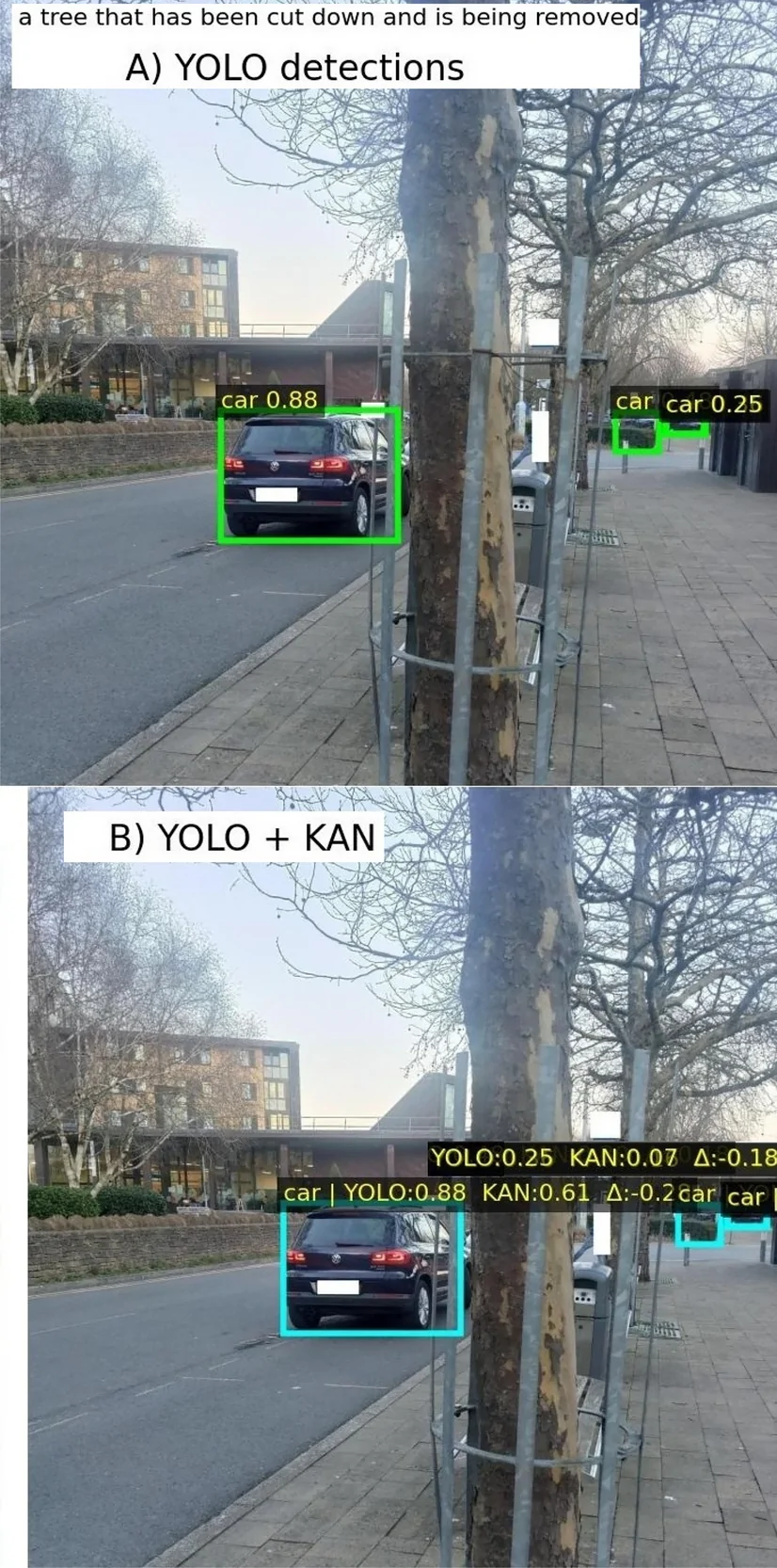
Application to a challenging scene with partial occlusion and blur.

**Fig. 22:**
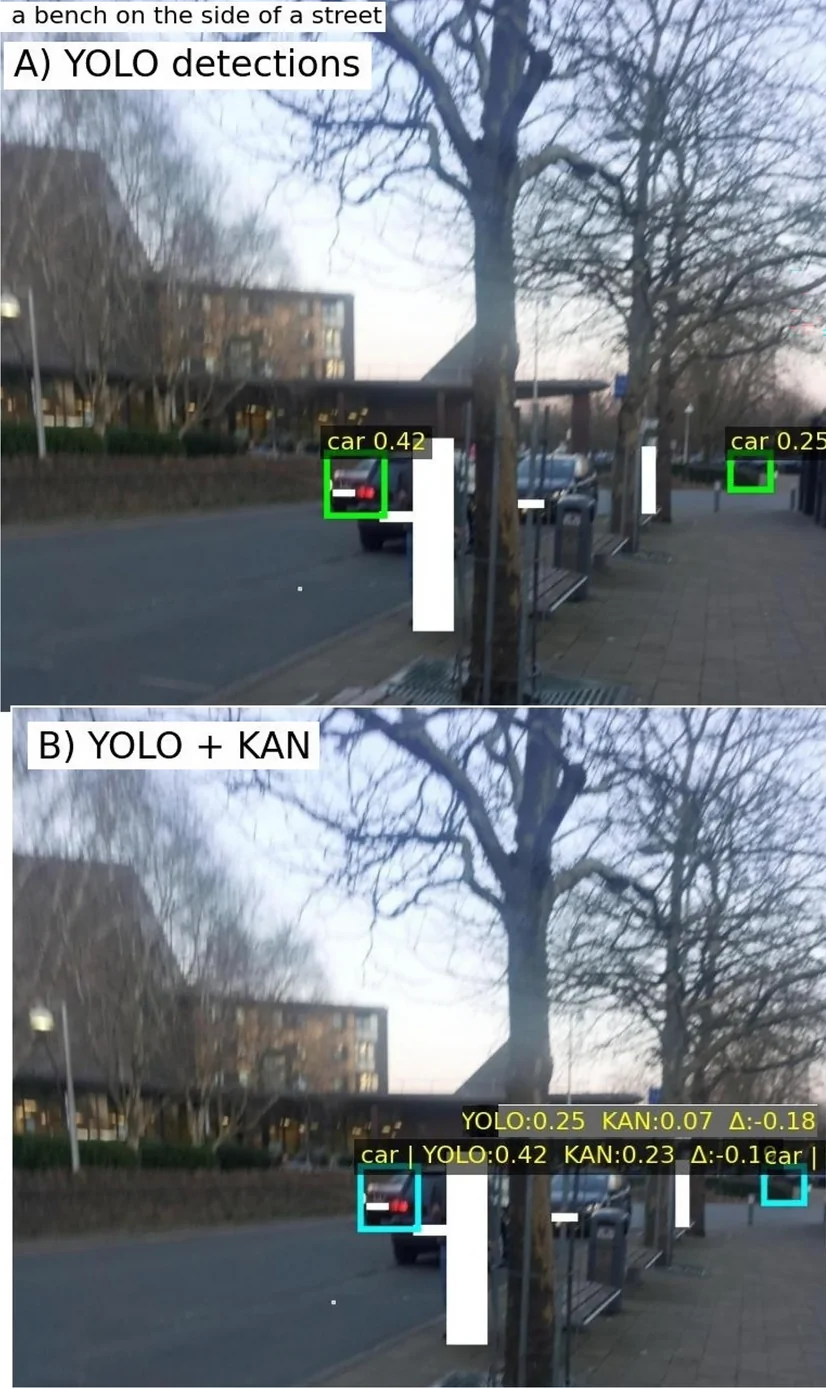
Application to a scene with multiple objects.

**Fig. 23:**
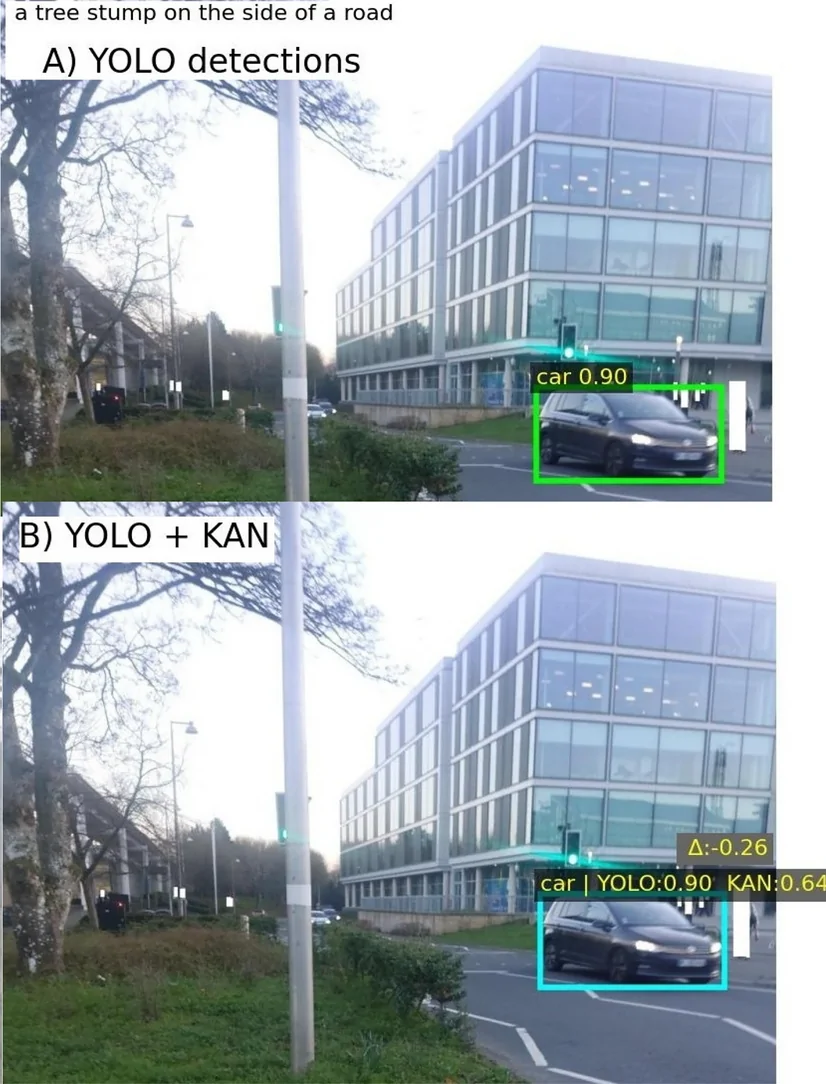
Application to a scene with unclear objects.

Figures 7, 8, 9, 10 present the partial dependence plots for individual features, demonstrating how the Kolmogorov-Arnold network output varies when only one input is changed. The dependence on bounding-box width (Fig. 7) shows a smooth monotonic increase, indicating that the model assigns higher confidence to wider objects. Height (Fig. 8) yields a similarly smooth but slightly weaker trend. The dependence on vertical position *y* (Fig. 9) is shallow with mild curvature, consistent with weak geometric sensitivity. Image scale (Fig. 10) exhibits only a small positive effect, confirming that detection confidence is not strongly biased across image resolutions. Across all plots, the curves are smooth and stable, illustrating that the spline components of the surrogate generalize without overfitting. Figures 11 and 12 show the feature-unit correlation bars. They provide fine-grained interpretability of the internal structure of the surrogate. The full input–hidden edge importance heatmap is also shown in Fig. 13, revealing that class and confidence supply strong signals to specific neurons, while geometric inputs connect more weakly. Figures 14, 15, 16 show the collection of spline functions learned by the Kolmogorov-Arnold network for all feature–unit pairs. These visualizations demonstrate the smoothness and diversity of the learned transformations. Each figure corresponds to one group of spline subplots. Finally, Figures 17, 18, 19, 20, 21, 22, 23 illustrate the application of the full system to real scenes. You Only Look Once detections are shown alongside Kolmogorov-Arnold network-predicted confidence estimates and vision-language captions. In high-quality images such as the truck (Fig. 17), police car (Fig. 18), and sand-car scenes (Fig. 19), the surrogate and detector agree closely. For more challenging examples featuring occlusions, blur, or clutter (Figs. 20, 21, 22, 23), the Kolmogorov-Arnold network highlights regions where confidence becomes less predictable, demonstrating the surrogate’s ability to expose when detection reliability decreases. The numerical interpretability complements the linguistic explanations generated by the vision-language model.

**Table 1:** Feature-level statistics from the network.

**Feature**	**SplineActivation**	**Saliency**	**PD Delta**
x	0.107281804	1.43$\times 10^{-6}$	*-0.007746*
y	0.106276410	2.64$\times 10^{-6}$	*-0.025138*
w	0.061890736	4.17$\times 10^{-6}$	0.056968
h	0.068972364	2.35$\times 10^{-6}$	*-0.045587*
conf	0.191350000	1.06$\times 10^{-4}$	0.706939
cls	3.731932000	1.67$\times 10^{-6}$	0.029724
scale	0.082707120	2.77$\times 10^{-6}$	*-0.0269816*

**Table 2:** Hidden-node statistics from the network.

**Node**	**Activation**	**Importance**	**Feature**	**Correlation**
n0	4.908808	2.2996383	cls	0.999684334
n1	3.8972838	0.19116642	cls	−0.999700487
n2	2.4302435	0.10419141	cls	−0.996881783
n3	6.031918	3.575461	cls	0.999689877
n4	5.654485	3.7371411	cls	0.999682188
n5	6.2070827	5.189023	cls	0.999588966
n6	5.103179	0.22900744	cls	−0.999275625
n7	0.48421517	0.27280924	cls	−0.947130859
n8	1.4790272	0.48364034	cls	−0.993801713
n9	0.6606282	0.2996985	conf	−0.958717942
n10	5.8533688	2.5290031	cls	0.999058664
n11	9.327825	0.38113362	cls	−0.999662876
n12	2.3340948	0.15058956	cls	−0.997766674
n13	0.4784846	0.076826476	cls	0.985139787
n14	2.45556	0.08944808	cls	−0.999845147
n15	2.6013832	1.7512177	cls	0.997587681

**Table 3:** Input-hidden feature influence values for each Kolmogorov-Arnold network hidden unit.

**Node**	**x**	**y**	**w**	**h**	**conf**	**cls**	**scale**
n0	0.029854016	0.015423812	0.002957478	0.014497110	0.046715240	2.295937500	0.005541976
n1	0.000579606	0.002248535	0.001634980	0.001924950	0.003963163	0.191089600	0.000456766
n2	0.000505880	0.002597063	0.002021160	0.002741036	0.005081795	0.103393555	0.000257480
n3	0.011441349	0.020771712	0.031916957	0.032681603	0.085905920	3.580443100	0.004258949
n4	0.023302530	0.028058290	0.026650915	0.024421094	0.055909153	3.746629200	0.022205167
n5	0.039337154	0.018811513	0.039905798	0.024799882	0.136980850	5.175523300	0.015163474
n6	0.003420651	0.000805297	0.001067619	0.001793040	0.006243926	0.227985070	0.002086347
n7	0.012705687	0.014479717	0.004435883	0.003144299	0.086433806	0.245138540	0.001393769
n8	0.006420781	0.012792237	0.009923225	0.006143517	0.056687247	0.489789370	0.000277586
n9	0.045870207	0.021616014	0.054148242	0.003510625	0.305599600	0.025778690	0.019549502
n10	0.021460332	0.017167496	0.017201278	0.029365148	0.095861495	2.544660300	0.007906381
n11	0.000112305	0.002232117	0.001292159	0.003263123	0.007255736	0.379950200	0.000122972
n12	0.002334908	0.001076487	0.000819806	0.001019987	0.009017214	0.148895730	0.000324138
n13	0.009948854	0.005338817	0.003623644	0.006526013	0.003195604	0.074584790	0.000295031
n14	0.000178230	0.001063958	0.000743822	0.000746206	0.000768538	0.089053730	0.000452965
n15	0.019759016	0.017285729	0.023938041	0.010737362	0.115140710	1.733505800	0.001825250

**Table 4:** Feature influence metrics combining spline activation, saliency, partial dependence delta, and edge importance.

**Feature**	**SplineAct**	**Saliency**	**PDP_Delta**	**EdgeImportance**	**Influence**
x	0.10728	2.86$\times 10^{-6}$	*-0.0077*	0.22723	0.01739
y	0.10628	5.29$\times 10^{-6}$	*-0.0251*	0.18177	0.01392
w	0.06189	8.34$\times 10^{-6}$	0.05697	0.22228	0.04231
h	0.06897	4.70$\times 10^{-6}$	*-0.0456*	0.16731	0.00371
conf	0.19135	2.12$\times 10^{-4}$	0.70694	1.02076	0.52001
cls	3.73193	3.33$\times 10^{-6}$	0.02972	21.05236	0.52559
scale	0.08271	5.55$\times 10^{-6}$	*-0.02698*	0.08212	0.01082

**Table 5:** Kolmogorov-Arnold network fidelity across overall data and per-feature quantile bins.

**Scope**	**Feature**	**BinIndex**	**BinRange**	**N**	**R2**	**MAE**	**RMSE**
Overall				9098	0.99568467	0.010857625	0.01510572
Per-Feature	x	0	[0.0078, 0.2581]	1820	0.995543874	0.010738888	0.014922408
Per-Feature	x	1	[0.2581, 0.4369]	1819	0.995584850	0.011135795	0.015436348
Per-Feature	x	2	[0.4369, 0.5569]	1820	0.995243065	0.011685996	0.016162014
Per-Feature	x	3	[0.5569, 0.7306]	1819	0.995711381	0.010715223	0.015072694
Per-Feature	x	4	[0.7306, 0.9956]	1820	0.996162495	0.010012295	0.013840625
Per-Feature	y	0	[0.0090, 0.3730]	1820	0.993737923	0.012518246	0.017162563
Per-Feature	y	1	[0.3730, 0.4995]	1819	0.996216900	0.010224011	0.014291467
Per-Feature	y	2	[0.4995, 0.5889]	1820	0.996546371	0.009600522	0.013792516
Per-Feature	y	3	[0.5889, 0.7164]	1819	0.995977398	0.010252580	0.014582687
Per-Feature	y	4	[0.7164, 0.9839]	1820	0.995056666	0.011692083	0.015466231
Per-Feature	w	0	[0.0078, 0.0633]	1820	0.994522331	0.009916774	0.013265161
Per-Feature	w	1	[0.0633, 0.1175]	1819	0.996003601	0.009420515	0.013088631
Per-Feature	w	2	[0.1175, 0.1942]	1820	0.996316109	0.009662537	0.013505891
Per-Feature	w	3	[0.1942, 0.3645]	1819	0.995564989	0.010814356	0.015195336
Per-Feature	w	4	[0.3645, 1.0000]	1820	0.992965135	0.014473127	0.019501280
Per-Feature	h	0	[0.0134, 0.0996]	1820	0.993620586	0.010833444	0.014169815
Per-Feature	h	1	[0.0996, 0.1767]	1819	0.994757525	0.011014964	0.014902389
Per-Feature	h	2	[0.1767, 0.2982]	1820	0.994644570	0.011212999	0.015743511
Per-Feature	h	3	[0.2982, 0.5189]	1819	0.994923902	0.011166411	0.015544278
Per-Feature	h	4	[0.5189, 1.0000]	1820	0.995195339	0.010060562	0.015118543
Per-Feature	conf	0	[0.2501, 0.3561]	1820	0.646103634	0.013054217	0.018254275
Per-Feature	conf	1	[0.3561, 0.5248]	1819	0.908017482	0.010815061	0.015045941
Per-Feature	conf	2	[0.5248, 0.7179]	1820	0.953721345	0.009243234	0.012182652
Per-Feature	conf	3	[0.7179, 0.8677]	1819	0.880090188	0.011291197	0.014700041
Per-Feature	conf	4	[0.8677, 0.9854]	1820	0.684149733	0.009884628	0.014724099
Per-Feature	cls	1	[0.0000, 3.0000]	3558	0.999025715	0.005320589	0.007107449
Per-Feature	cls	2	[3.0000, 29.0000]	1878	0.992935624	0.014938466	0.019866999
Per-Feature	cls	3	[29.0000, 52.0000]	1833	0.992111145	0.014669338	0.018721599
Per-Feature	cls	4	[52.0000, 79.0000]	1829	0.994633838	0.013618740	0.016740484
Per-Feature	scale	0	[0.0745, 0.6250]	1800	0.995370806	0.011333028	0.015715895
Per-Feature	scale	1	[0.6250, 0.6672]	1303	0.995758532	0.010512075	0.014713371
Per-Feature	scale	2	[0.6672, 0.7375]	2343	0.995833673	0.010658501	0.014828051
Per-Feature	scale	3	[0.7375, 0.7500]	225	0.995384340	0.011505265	0.015818063
Per-Feature	scale	4	[0.7500, 1.0000]	3427	0.995732202	0.010832923	0.015066167

**Table 6:** Monotonicity analysis of each input feature.

**Feature**	**MonotonicityScore**	**Direction**	**Strength**
x	0.021862028	Flat/Weak	Weak
y	0.034401234	Flat/Weak	Weak
w	0.376517522	Positive	Moderate
h	0.449024012	Positive	Moderate
conf	0.996658272	Positive	Strong
cls	−0.146067093	Negative	Weak
scale	0.022657916	Flat/Weak	Weak

**Table 7:** A quantitative trust assessment scoring system using the KAN-YOLO confidence discrepancy (*Δ*) and BLIP captions.

**Image**	**YOLO**	**KAN**	$\boldsymbol{\Delta }$	**Decision**	**BLIP Caption**
Fig. 17	0.86	0.86	0.00	Accept	A red truck parked in front of a house
Fig. 18	0.90	0.90	0.03	Accept	A police car parked on the side of the road
Fig. 19	0.91	0.89	0.02	Accept	A man is standing next to a car in the sand
Fig. 20	0.94	0.93	0.01	Accept	A white van with a blue and yellow design
Fig. 21	0.88	0.61	0.27	Consider	A tree that has been cut down and it is being removed
Fig. 22	0.42	0.23	0.19	Review	A bench on the side of a street
Fig. 23	0.90	0.64	0.26	Consider	A tree stump on the side of a road

Decision rule: Accept ($\Delta \le 0.05$), Review ($0.05 < \Delta \le 0.25$), Consider (*Δ> 0.25*).

**Table 8:** Sensitivity analysis of the YOLO features for the KAN predictions, with indicated importance of vision-language captions for human interpretation and intervention.

**Experiment**	**R2**	**MAE**	**BLIP Importance**
Full model (7 features)	0.996	0.0109	Case dependent
Remove x	0.995	0.0116	Case dependent
Remove y	0.994	0.0141	Case dependent
Remove w	0.995	0.0116	Case dependent
Remove h	0.995	0.0122	Case dependent
Remove conf	−5.103	0.518	High
Remove cls	0.996	0.0057	Case dependent
Remove scale	0.992	0.0157	High

**Table 9:** Runtime analysis of YOLO, KAN, and the combined system.

**Component**	**Mean (ms)**	**Std (ms)**	**Description**
YOLOv10	174	383	Baseline detector
KAN	15	6	Interpretability module
YOLO+KAN	215	446	Combined system
*Overhead: 41 ms relative to YOLO inference time*			

Table 1 summarizes the per-feature statistics extracted from the Kolmogorov-Arnold network. The mean spline activation identifies class and confidence as the dominant drivers of the model’s confidence behavior, while the partial dependence deltas quantify each feature’s global effect across its domain. Geometric inputs play a secondary role, and the uniformly small saliency values demonstrate that the network remains smooth and stable. Table 2 provides a detailed description of hidden-unit behavior within the Kolmogorov-Arnold network. Strong correlations between hidden-unit activations and the class feature reveal a consistent specialization pattern, with a few neurons carrying disproportionately large influence over the output. This structure supports a transparent interpretation of the model, where individual units serve identifiable semantic functions rather than mixing signals in an opaque manner. Table 3 presents the complete input–hidden edge importance matrix computed from spline coefficients. The results show strong connections from class and, to a lesser extent, confidence, feed into specific hidden units, while geometric features contribute weaker. Table 4 consolidates all interpretability metrics into a unified feature-influence score. The strong agreement between normalized statistics confirms that class and confidence dominate the model’s confidence landscape from multiple perspectives; activation strength, global effect, and structural edge contribution. This agreement reinforces the internal coherence of the network and provides a robust grounding for the qualitative partial dependence plots analyses. Table 5 evaluates the Kolmogorov-Arnold network fidelity across quantile bins for each feature. The model maintains stable fidelity across most of the feature space, demonstrating that the additive spline formulation accurately captures the functional relationship between the model’s confidence and its input parameters. The slight reductions in fidelity for the lowest and highest confidence bins align with the expected difficulty of modeling edge-case detections. Finally, Table 6 shows the monotonicity scores, offering a global characterization of the directionality of the model’s confidence behavior. In addition, Table 7 extends the qualitative analysis by introducing a quantitative trust assessment scoring system based on the discrepancy between YOLO and KAN confidence scores. The defined threshold-based categorization (accept, review, consider) provides a mechanism which transforms visual comparisons into actionable outcomes. Table 8 presents the sensitivity analysis evaluating the contribution of individual input features and spline configurations to the KAN model. The geometric features (x, y, w, h) and image scale exhibit only a marginal influence, with minimal degradation observed upon their removal. Interestingly, excluding the class feature slightly improves performance, confirming that its apparent importance arises from its numerical encoding rather than meaningful semantic contribution. Table 9 presents a quantitative runtime analysis. The proposed framework maintains consistent performance, with the increased variance primarily driven by the image-dependent complexity of the detection stage.

## Discussion

The interpretability analysis produced by the Kolmogorov-Arnold network reveals several consistent patterns which require discussion for further potential applications^115–127^. The unified feature influence table is the most essential as it captures both the spline-activation behaviour and the structural edge importance values, effectively integrating the core components. The results show that the surrogate processes each input feature through dedicated spline transformations, highlighting the interpretability advantage of additive univariate nonlinearities.

Importantly, unlike geometric features, image-quality descriptors are not implicitly represented in the input space. However, their influence is already embedded within the YOLO confidence score, which reflects detection reliability under varying visual conditions, thereby eliminating the need for explicit inclusion.

The reduced fidelity observed at the lowest and highest confidence bins in Table 5 can be attributed primarily to data distribution effects rather than limitations of the framework. Low-confidence detections inherently exhibit higher uncertainty and noise, making their behavior more difficult to approximate, while high-confidence samples are relatively sparse, reducing the effective training signal in this region.

Importantly, the class index is treated as a scalar input in the model. This provides a compact representation of class-conditioned variations in detection confidence, which are then approximated using smooth spline functions. Here, the sensitivity study in Table 8 shows that removing the class feature does not degrade performance and can even slightly improve it. Embedding-based encodings could be explored in future work to better represent categorical information.

## Conclusion

This work presented a self-aware framework that enhances the transparency and trustworthiness of You Only Look Once detections through an interpretable Kolmogorov-Arnold network. The visual-language captioning component assisted the perception pipeline with lightweight multimodal descriptions. The framework provides an interpretable approach with:

1. Numerical insight into whether You Only Look Once’s confidence values are trustworthy.
2. Real-time and practical provision of confidence across the full feature domain, while simultaneously identifying unreliable detections in visually ambiguous scenes where the detector may fail.
3. Analysis of hidden units and monotonicity patterns for a coherent internal structure, with specialised neurons that consistently encode class information, confidence trends, and interpretable geometric influences.
4. A multimodal extension using vision-language descriptive scene-level context.

Importantly, the findings show that the proposed framework enables detection with more reliable confidence estimation, strengthening the transparency and robustness of modern automated systems.

## Methods

You Only Look Once is a one-stage object detector. Given an input image $I \in \mathbb {R}^{H \times W \times 3}$, the model computes a hierarchy of feature maps through a backbone network, producing multiscale feature tensors $[ F_1,F_2,F_3 ]$ that encode semantic and spatial information. At each spatial location on each feature map, the model predicts a bounding box parameterized by its center and dimensions:

1 $$\begin{aligned} b = (x,\, y,\, w,\, h) \end{aligned}$$

together with a score *o ∈ [0,1]* and a distribution over classes *p*(*c*|*I*). The final confidence for a detection is computed as:

2 $$\begin{aligned} \textrm{conf} = o \cdot p(c|I) \end{aligned}$$

which serves as the scalar quantity for the Kolmogorov-Arnold Network. The network receives an input image of size $640 \times 640 \times 3$, which is processed by the backbone network to extract hierarchical visual features. The backbone primarily consists of convolutional layers and coarse-to-fine modules that progressively learn spatial and semantic representations from the input image. These extracted features are then passed to the neck component to aggregate multi-scale contextual information and enhance feature fusion. The refined feature maps are subsequently forwarded to the detection head, before the model produces a set of dense predictions corresponding to potential object locations and class probabilities across the image, resulting in approximately 8400 candidate detections.

Let $(\hat{x},\hat{y},\hat{w},\hat{h})$ denote the network’s predicted box and $(x^{*},y^{*},w^{*},h^{*})$ denote the ground truth box assigned to that feature-map location, the model employs a differentiable intersection over union (IoU) metric-based loss function as:

3 $$\begin{aligned} L_{\textrm{box}} = 1 - \textrm{IoU}\!\left( (\hat{x},\hat{y},\hat{w},\hat{h}),\,(x^{*},y^{*},w^{*},h^{*})\right) \end{aligned}$$

The training objective is expressed as:

4 $$\begin{aligned} L = \lambda _{\textrm{box}} L_{\textrm{box}} + \lambda _{\textrm{obj}} L_{\textrm{obj}} + \lambda _{\textrm{cls}} L_{\textrm{cls}} \end{aligned}$$

where $\lambda _{\textrm{box}},\lambda _{\textrm{obj}},\lambda _{\textrm{cls}}$ are balancing coefficients. Each detection consists of the tuple $(x,y,w,h,\textrm{conf},c)$, from which seven numerical features are used by the Kolmogorov-Arnold network. These are the normalized spatial position (*x*, *y*), the normalized size (*w*, *h*), the predicted confidence $\textrm{conf}$, the class index *c*, and the relative image scale $s = \tfrac{wh}{640^{2}}$.

The Kolmogorov-Arnold network is inspired by the Kolmogorov-Arnold representation theorem, which states that any multivariate continuous function $f:\mathbb {R}^n \rightarrow \mathbb {R}$ can be expressed as a finite superposition of univariate continuous functions. Kolmogorov proved that there exist continuous functions $\phi _q$ and $\psi _{p,q}$ such that:

5 $$\begin{aligned} f(x_1,\ldots ,x_n) = \sum _{q=1}^{2n+1} \phi _q\!\left( \sum _{p=1}^{n} \psi _{p,q}(x_p) \right) \end{aligned}$$

Kolmogorov–Arnold networks operationalize this idea by replacing the learned $\psi _{p,q}$ functions with trainable spline functions $s_{j,k}(\cdot )$ to every connection between input feature *k* and hidden unit *j*, and replacing $\phi _q$ with linear combinations of hidden-unit activations as:

6 $$\begin{aligned} h_j(x) \;=\; \sum _{k=1}^{d} s_{j,k}(x_k) \end{aligned}$$

where *d* is the input dimensionality. The model output is then obtained as:

7 $$\begin{aligned} y(x) \;=\; \sum _{j=1}^{m} w_j\,h_j(x) \end{aligned}$$

where *m* is the number of hidden units and $w_j$ are learned output weights. The model input signals are fed into a hidden layer in which each neuron applies a learnable univariate spline function, shown in the diagram as a white spline curve inside each hidden unit. The outputs of the hidden spline neurons are subsequently combined by a standard output neuron to produce the final prediction.

In this work, the surrogate confidence model is a Kolmogorov-Arnold network with architecture width=[7,16,1], grid=5 and spline order=3 (cubic b-splines), meaning that the network receives seven interpretable numerical features derived from detections. These features consist of normalized bounding–box geometry (*x*, *y*, *w*, *h*), predicted confidence, discrete class index, and relative image scale $(wh / 640^2)$. Finally, the model learning form is:

8 $$\begin{aligned} \widehat{c}(x) = \sum _{j=1}^{16} w_j \left( \sum _{k=1}^{7} s_{j,k}(x_k) \right) \end{aligned}$$

where $x \in \mathbb {R}^7$ is the vector of You Only Look Once-derived features. This architecture directly enables the interpretability results. Partial-dependence plots arise naturally from evaluating a single spline while holding other dimensions fixed. Specifically, monotonicity emerges from the smoothness properties of the fitted splines, hidden-unit roles can be inferred from correlations between spline outputs and input features, and input-hidden edge importance corresponds to norms of learned spline coefficients. Importantly, each input–hidden connection uses a learnable spline made from B-spline basis functions with grid = 5 and spline order = 3, each with its own trainable coefficient. During training, these coefficients adjust the curve shape, enabling smooth and interpretable nonlinear mappings while keeping the model simple and stable.

Large vision-language foundation models aim to learn a joint representation between images and natural language. An input image *I* and a textual sequence $T=(t_1,\ldots ,t_L)$ are embedded into a unified representation followed by a joint conditioning module:

9 $$\begin{aligned} H = F_{\textrm{VL}}\bigl (Z_{\textrm{img}}, Z_{\textrm{txt}}\bigr ) \end{aligned}$$

where, $F_{\textrm{VL}}$ consists of stacked transformer blocks, and $Z_{\textrm{img}}$ and $Z_{\textrm{txt}}$ are the unified representations of the input. The generative language model then predicts a probability distribution over the vocabulary conditioned jointly on vision and text as:

10 $$\begin{aligned} p(t_{i+1}\,|\,t_1,\ldots ,t_i,I) = \textrm{Softmax}\!\left( W\,h_i \right) \end{aligned}$$

where, $h_i$ is the decoder hidden state produced from the fused representation *H*. The input image is first processed by the processor, which performs the required tokenisation, normalisation, and embedding transformations. These processed embeddings are then passed to a vision encoder and a text decoder, which jointly generate a natural-language caption describing the visual content of the image.

Bootstrapped language-image pretraining optimizes a contrastive objective of the form:

11 $$\begin{aligned} L_{\textrm{ITC}} = -\log \frac{ \exp \!\left( \textrm{sim}\bigl (Z_{\textrm{img}},Z_{\textrm{txt}}\bigr )/\tau \right) }{ \sum _{T'} \exp \!\left( \textrm{sim}\bigl (Z_{\textrm{img}},Z_{\textrm{txt}}'\bigr )/\tau \right) } \end{aligned}$$

where $\textrm{sim}(\cdot ,\cdot )$ is a cosine-similarity operator and *τ* is a temperature parameter. The model functions as a language-based interpretability layer that complements the numerical explanations provided by the Kolmogorov-Arnold network. For each detection image *I*, BLIP receives the visual input and produces a caption $\hat{T}$ by sampling from the conditional distribution *p*(*T*|*I*) given by the decoder.

## Data Availability

The data that support the findings of this study are available from publicly accessible sources and locally collected datasets. The Common Objects in Context (COCO) dataset is publicly available. Additional image data collected from the University of Bath campus are available from the corresponding author upon reasonable request.

## Code availability

The code used to support the findings of this study is available from the corresponding author upon reasonable request.
